# Attitudes towards COVID-19 Booster Vaccines, Vaccine Preferences, Child Immunization, and Recent Issues in Vaccination among University Students in Jordan

**DOI:** 10.3390/vaccines10081258

**Published:** 2022-08-04

**Authors:** Soukaina Ryalat, Hamza Alduraidi, Saif Aldeen Al-Ryalat, Marah Alzu’bi, Muntaser Alzyoud, Nada Odeh, Jawad Alrawabdeh

**Affiliations:** 1 Department of Oral Surgery, Oral Medicine, Oral Pathology, Periodontics and Oral Radiology, Faculty of Dentistry, The University of Jordan, Amman 11942, Jordan; 2 School of Nursing, The University of Jordan, Amman 11942, Jordan; 3 School of Medicine, The University of Jordan, Amman 11942, Jordan

**Keywords:** COVID-19, booster vaccines, child immunization

## Abstract

Although COVID-19 vaccines have been available in Jordan for more than a year, Jordan suffers from a low vaccination rate. The aim of this study was to explore attitudes towards recent issues in vaccination among university students in Jordan. We adopted a cross sectional study design using an online questionnaire distributed in a Jordanian university with a medical school chosen at random. The survey asked about COVID-19 vaccine preferences, factors affecting COVID-19 vaccine preferences, child vaccination, and booster vaccines. A total of 417 students completed the survey. Most respondents (54.7%) preferred the Pfizer vaccine, and 6.2% refused to take any vaccine. Pfizer’s efficacy against new strains is a main factor in preferring Pfizer over other vaccines (*p* < 0.01). Most respondents (71%) believed that vaccination is crucial to prevent COVID-19 surges from new COVID-19 strains, while 44.6% of respondents believed that children should be included in vaccination campaigns, and 70% believed that booster vaccines required more studies to prove their efficacy. Students had mixed attitudes towards many recent issues concerning COVID-19 vaccination. Studying these factors and attitudes in more depth and in different populations can pave the way towards improving vaccination rates worldwide.

## 1. Introduction

The coronavirus-19 disease, COVID-19, was declared a global pandemic by the World Health Organization (WHO, Geneva, Switzerland) on 11 March 2020 [1]. This virus has taken a heavy toll on the health and economies in all countries around the globe, especially in developing countries. These countries were particularly not as prepared due to inadequate workforces and capabilities in the field of medical care, which put them at high risk of healthcare system collapse.

Before the development of vaccines, many non-medical interventions were initially adopted to fight the disease, such as social distancing, regular quarantines, and restrictions on public gatherings. However, these are still less effective in limiting the spread of infections compared to vaccines. They also pose great burdens on the levels of the global economy and social wellbeing, thus reducing quality of life and increasing costs [2,3,4]. Simultaneously, global collaborative efforts were made to ensure the manufacturing of a safe and efficient COVID-19 vaccine as quickly and safely as possible [5]. Unfortunately, many individuals have grown skeptical of the effectiveness and safety of the newly developed vaccines due to a variety of reasons such as religious beliefs, conspiracy beliefs, claims of a lack of evidence, and worry that vaccine efficacy would decrease with the emergence of novel strains. Social media platforms further facilitated the propagation of these beliefs which hindered the enforcement of protective health measures resulting in highly variable levels of compliance by the public.

Global vaccination efforts started ever since the Food and Drug Administration (FDA) issued the first Emergency Use Authorization (EUA) for the Pfizer-BioNTech COVID-19 vaccine on 11 December 2020. However, concerns about waning vaccine induced immunity and the emergence of new coronavirus variants sparked considerable debate regarding the need for COVID-19 vaccine booster shots. Most variants of concern shared a similar recurring theme of mutations in the receptor-binding domain of the spike protein. These mutations were associated with increased recurrence of infection, transmissibility, and immune evasion even after natural immunity caused by infection or vaccine induced immunity [6,7,8,9]. Booster doses were shown to provide a fast and significant improvement in protection against infection by all variants as they improved the humoral and cellular immunity against different variants [10,11].

In the earlier periods of the COVID-19 pandemic, children comprised a smaller subset of significant infections since the majority of infected children were asymptomatic or only had mild symptoms. Despite that, children still comprise a major part of society and their vaccination is a crucial step to achieving herd immunity. Recent data show that child infections by COVID-19 were on a steady rise, displaying a sharp and sudden increase in severity and transmission among children, which correlated with the emergence of the COVID-19 variants of concern. These variants had higher breakthrough infection capabilities [12,13,14]. Parents and guardians are the main decision makers of their children’s vaccination, and they have the right to understand the risks and benefits of vaccination. Educating them about variants and their increased risk to children may drive them to vaccinate their children despite any hesitancy they might have.

Jordan is one of the youngest countries in the world, with 63% of its population being under the age of 30 [15]. Due to that, we aimed to study specific vaccine preferences and factors affecting vaccine preferences, hesitancy towards COVID-19 vaccine boosters, attitudes towards child vaccination, and other recent issues in vaccination such as the importance of vaccination against new COVID-19 strains in a population of university students. Our goal is to better understand this population to tailor awareness and educational programs, address most critical issues inducing hesitancy in the population as a whole, and leverage the education of the young to further convince the elderly to get vaccinated.

## 2. Methodology

### 2.1. Study Design and Settings

We adopted a cross-sectional study design to study COVID-19 vaccine preferences and attitudes towards child immunization, booster vaccines, and new COVID-19 strains among Jordanian university students. There are 31 universities in Jordan, only 6 of which have medical schools, and by random selection we chose one of these universities. We stratified the university’s population into 3 strata depending on their school (medical school, non-medical health school, and non-health schools). Each stratum in our sample included about 130 students who were chosen by convenience sampling. The study was done using a questionnaire developed on Google Forms. The questionnaire was distributed online by posting on each batch’s group on Facebook, and by sending it to friends and colleagues. Our inclusion criteria included any undergraduate student with an internet connection in the chosen university. Our exclusion criteria excluded any post graduate students (masters or Ph.D. students), faculty members, and other workers in the chosen university.

### 2.2. Instrument Design and Validity

We developed an online self-administered questionnaire on Google Forms. The questionnaire was developed in English, translated into Arabic, and reviewed to make sure it was consistent in meaning in both languages. Participants had the ability to choose the language they preferred to complete the questionnaire in.

The questionnaire started with a paragraph explaining the intent of the study, the requirement of informed consent, and assured the participants that all data would be treated confidentially, with no harm to participants. The second section of the questionnaire was the demographics section, which asked participants about demographic factors such as gender, age, GPA, year of study, and school category. This section also asks participants about their attitudes towards new COVID-19 strains, with five possible answers being: ‘COVID-19 variant strains aren’t that dangerous and shouldn’t be given much attention’, ‘COVID-19 variant strains are dangerous only for the elderly or people with chronic diseases’, ‘No difference between the original strain and the new strains’, ‘COVID-19 variant strains can be potentially dangerous and should be dealt with properly’, COVID-19 variant strains are very dangerous and highly deadly’. Participants were also asked four questions about recent issues in vaccination, specifically asking about attitudes towards child immunization, the importance of vaccination in preventing a new COVID-19 wave by new strains, the effect of hearing about new COVID-19 strains on the desire for vaccination, and their thoughts about the effect of the emergence of new strains on the efficacy of the available vaccines. The answers to all aforementioned questions followed a 5-point Likert scale format (strongly disagree, disagree, neutral, agree, strongly agree). Participants were also asked whether they believe citizens should be able to choose what vaccine they prefer to choose (answers being yes/no), and their opinions on booster vaccines (answers being yes/no/I’m waiting for more studies).

Participants were then asked about what vaccine they would prefer taking from five current vaccines (Pfizer, AstraZeneca, Sputnik V, Johnson and Johnson, and Sinopharm). Additional options were available such as (‘I don’t mind any vaccine’, ‘any vaccine other than Sinopharm’, ‘any vaccine other than mRNA vaccines’, and ‘I prefer no vaccine’). Participants were also asked to choose what factors affect their vaccine choice from a number of factors such as the perceived increased efficacy of the Pfizer vaccine against new strains, trust in WHOs standards, perceived lower efficacy of certain vaccines, harmful side effects of certain vaccines, ease of travel after taking certain vaccines, herd immunity, belief that COVID-19 vaccines are unsafe, and harmful side effects of mRNA vaccines.

### 2.3. Pilot

We conducted a pilot study on 28 students and received both quantitative and qualitative feedback from the respondents. The questionnaire was edited according to the feedback received to improve the clarity and brevity of the questions.

### 2.4. Data Collection

Our self-structured questionnaire was distributed online by posting on each major batches’ group on Facebook, and by sending it to friends and colleagues. Our inclusion criteria included any student with an internet connection in the chosen university. Responders were 417 students who participated in the study voluntarily and anonymously.

### 2.5. Ethical Approval

This study was approved by the IRB (Institutional Review Board) at the University of Jordan hospital. For participants’ consent, an information sheet was added to the questionnaire with all the details participants need to know in order to decide whether to participate or not.

### 2.6. Data Analysis

Data analysis was carried out using IBM SPSS Statistics for Windows, Version 27.0. Armonk, NY, USA: IBM Corp. Descriptive statistics such as means, medians, and frequencies were used to describe the sample. ANOVAs and *t*-tests were used to compare means between multiple groups and two groups, respectively. Chi square tests were used to find relationships between categorical variables, then adjusted standardized residuals were used for post-hoc testing. Phi and Cramer’s v were used as effect size measurements for chi square analyses. Fisher’s exact tests were used instead of chi square tests when chi square assumptions were violated.

## 3. Results

### Sociodemographic Characteristics

417 people completed the questionnaire. The mean age for the participants was 20.14 (SD = 1.683). Most of the participants were female (69.3%). Participants were distributed roughly equally among the three school categories, and close to half of the participants were in their second year (46%). Characteristics of the participants are shown in Table 1.

## 4. Vaccine Preferences

We asked participants to choose their preferred vaccine from five vaccines currently offered in Jordan, in addition to other options such as “I wouldn’t take any vaccine” or “I would take anything except mRNA vaccines”. The most popular vaccine was Pfizer (54.7%) followed by “I don’t mind any vaccine” (19.4%). 6.2% of participants stated that they wouldn’t take any vaccine. A chi-square analysis showed that vaccine preferences varied by school category (*p* = 0.001, Cramer’s V = 0.183), as medical students and non-medical health students were more likely to choose “any vaccine” compared to non-health students. Vaccine preferences didn’t vary significantly between genders (*p* > 0.05). All answers and percentages are shown in Table 2.

## 5. Factors Affecting Vaccine Preferences

**Figure 1:**
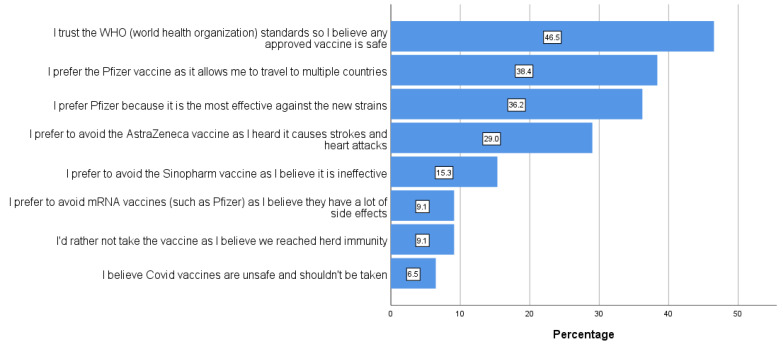
Factors affecting vaccine preferences.

Out of a total of eight factors (shown in Figure 1), almost half of the participants stated that they trusted the WHO recommendations. Interestingly, almost 40% of participants preferred Pfizer for its increased efficacy and its ability to facilitate travel to other countries that require vaccination, but don’t accept vaccines such as Sinopharm. 6.7% of participants believe that COVID-19 vaccines are unsafe. All factors and percentages are shown in Figure 1.

Pfizer’s effectiveness against new strains was a major factor for preferring Pfizer over other vaccines. Participants who trusted the WHO standards were significantly more likely to prefer any vaccine. Another factor that made participants prefer Pfizer was that the Pfizer vaccine made travelling to other countries easier. Nine percent of participants believed that mRNA vaccines have a lot of side effects, and so prefer avoiding them. More than six percent of participants believed that COVID-19 vaccines were not safe and should not be taken.

Chi square tests were used to find associations between each specific factor and vaccine choices, and Cramer’s V was used to determine the effect size. Participants who chose ‘I prefer Pfizer because it is the most effective against new strains’, ‘I prefer to avoid the Sinopharm vaccine as I believe it is ineffective’, or ‘I prefer the Pfizer vaccine as it allows me to travel to multiple countries’ were significantly more likely to prefer the Pfizer vaccine (*p* < 0.0001 for all 3 tests). Participants who chose ‘I trust the WHO standards so I believe any approved vaccine is safe’ were significantly more likely to prefer any vaccine (*p* < 0.0001), and participants who chose ‘I believe COVID vaccines are unsafe and shouldn’t be taken’ were significantly more likely to not take the vaccine (*p* < 0.0001).

All associated factors and details of the analyses are shown in Table 3.

## 6. Attitudes towards Recent Issues in Vaccination

Booster vaccines and the ability to choose preferred vaccines.

We asked participants if they agree that vaccine choice should be left to the citizens. An overwhelming number, 85.4%, agreed. In addition, 70% of participants stated that they were not sure whether booster vaccines are effective until more studies were conducted.

Females were more likely to agree that citizens should be able to choose their preferred vaccine, while the opposite was true for males (*p* < 0.001, phi = 0.181). 

Vaccination and new COVID-19 strains.

Participants were asked to state their level of agreement on four questions about recent issues related to vaccination. All questions and answer percentages are shown in figure two. Roughly half of the participants (44.6%) believed that children should be included in vaccination campaigns. The majority of participants (71%) believed that vaccination is crucial to avoid a new wave from the new COVID-19 strains. 

A Kruskal-Wallis test showed that medical students were more likely to answer question 3 positively compared to students from other school categories (adjusted *p* < 0.001). There were no significant differences between non-medical health students and non-health students’ answers. See Table 4.

## 7. Discussion

As a middle-income country with both considerable economic and health-related challenges, Jordan has given its all fighting the recent COVID-19 pandemic. A number of strict hygienic controls and social distancing measures imposed by the government helped greatly in overcoming the fiercest waves of the pandemic. Nevertheless, it is vaccination that had the ultimate important role in putting an end to those waves. According to the UNHCR, 29 main vaccination centers and 45 secondary centers operated across the Kingdom to provide the vaccine. Regardless, Jordan is still considered one of the lowest countries in terms of the uptake of COVID-19 vaccines [16]. Many Jordanians are still skeptical about vaccination, and conspiracy theories are widely spread in Jordanian society [17]. The implementation of mass-vaccination programs has to be accompanied by high levels of compliance to these programs and to other non-pharmaceutical hygienic and social measures for it to provide the desired protection against SARS-CoV-2 [18]. A 2021 Italian study discussed the issues that interfere with the success of mass-vaccination campaigns. The possible adverse effects of the vaccines, the lack of trust in COVID-19 vaccines’ efficacy, and the hesitancy that arose as a result of the impact of the COVID-19 pandemic on everyone’s mental health all impeded the flow of mass-vaccination programs and lowered people’s acceptance of the vaccine despite its benefits [19]. Very similar reasons were behind the low vaccination rates and the displeasing levels of compliance to mass-vaccination programs in Jordan [16]. With that being said, we conducted this study aiming to increase the vaccination rate, compliance to mass-vaccination programs, and people’s awareness about vaccination by going the extra mile in vaccination-related research through investigating what’s beyond vaccine acceptance and vaccine hesitancy, which are heavily studied [16,17,20]. These include vaccine preferences, factors affecting vaccine preferences, how the emergence of new COVID-19 strains affected vaccination acceptance, and—for the first time—people’s thoughts on children’s vaccination and boosting vaccine shots.

The following findings can be drawn from the present study. First, when asked about their vaccine preferences; approximately half of the participants chose Pfizer. Less than 10% of the participants stated that they wouldn’t take any vaccine. Accordingly, it would be safe to say that the vast majority of our study population lean towards taking the vaccine. This finding is inconsistent with the finding of a 2021 paper that showed that the acceptance of COVID-19 vaccines among young Jordanian adults is limited [21]. Second, almost 47% of the participants willing to take the vaccine justified this will by having faith in WHO standards, which made them believe that any WHO approved vaccine is safe. This finding is in harmony with the finding of a 2022 study in a western country in which 61% of participants believed in the safety of COVID-19 vaccines [22].

Additionally, a considerable percentage of participants leaning towards getting vaccinated favored Pfizer for its high efficacy and its ability to facilitate travel to numerous countries. This pattern of results is inconsistent with the results of a previously published Pakistani study, as 43% of its participants preferred Sinopharm over both Pfizer and AstraZeneca [23]. On the other hand, our results showed consistency with the results of an Iraqi study which revealed that Pfizer is the most preferred COVID-19 vaccine among its population [24]. It is noteworthy that only a minute percentage of our study participants believe that COVID-19 vaccines are unsafe and consequently refuse getting any. Third, we asked participants about their thoughts on the vaccination of children, as they are gradually becoming major players in this global pandemic. Interestingly, roughly half of the participants believed that children should be included in vaccination campaigns. This finding is similar to the finding of a recent study in the US in which approximately 50% of the participants indicated their acceptance of child COVID-19 immunization [25].

However, the acceptance of child COVID-19 immunization was fairly high in a new Chinese study (72.6%) [26]. Fourth, almost two thirds of the study population agreed with the fact that vaccination is crucial to avoiding any new waves of the pandemic that might arise from new variant COVID-19 strains. Fifth and last, 70% of the participants doubted the efficacy of booster vaccine shots and believed that more studies should be conducted in order for them to feel more certain about their necessity. This level of hesitancy is higher than that of Al-Qerem et al.’s (2022) work in which 45% of the participants expressed that they are willing to get the COVID-19 booster dose [27]. Additionally, around 50% of the participants of a 2021 study conducted in Jordan had no problem with receiving the COVID-19 booster shot [28]. Based on the results of the aforementioned studies, the level of hesitancy associated with COVID-19 booster shots among Jordanians reported by our study can be considered to be one of the highest levels, if not the highest.

Aligning with our previous finding, a study conducted in Hong Kong has also found studying medicine/health-related majors to be a contributing factor when it comes to vaccine preferences [29]. The choice of vaccine, however, varied drastically amongst different populations. Similar to our sample, some studies demonstrated higher acceptance of Pfizer as the vaccine of choice for the majority [30]. On the other hand, a recent study suggested that political views and conspiracy theory beliefs could play major roles in repelling against m-RNA based vaccines, and promoting for others [31].

Although half of our participants indicated their acceptance of child immunization, a considerable number were still uncertain. This could be related to concerns regarding both the safety and effectiveness the vaccines could actually provide [32]. Other explanations could include worries about subsequent complications, such as multisystemic inflammatory syndrome in children (MIS-C), a syndrome with symptoms similar to those of Toxic Shock Syndrome, as many cases were reported around the world [33]. Heterogeneity was found among caregivers and parents in other countries in terms of willingness and intention to vaccinate their children. Demographic factors were found to have a major contribution to the different perspectives [34,35]. Proper education and awareness of the vaccine’s efficacy could be utilized to help individuals make an informed decision.

The issue of booster shots was one with a question mark imposed on it, due to the lack of literature and RCTs previously. Perhaps the fact that 70% of our participants expressed skepticism towards the third shot could be attributed to the aforementioned point. On a more positive note, more studies and trials concerning the efficiency of booster doses have been published recently, which could be employed to enlighten the population regarding that matter. As it was confirmed by a clinical trial that booster shots following two doses of Pfizer or AstraZeneca do indeed boost immunological defense by enhancing both types of immunity [36]. The said trial, however, suggested that the availability of third doses would improve administration rates, unlike our sample that required more studies to be conducted to form an opinion.

Our findings highlight the essential need for the remodeling of public health strategies and the effective redirection of interventions in Jordan. Moreover, our findings highlight the essential need to invest in the present-day strategies used to promote COVID-19 vaccines, as they are not inclusive enough. Based on our study, increasing trust in WHO by the public could salvage many vaccination related issues, such as perceptions of the vaccination of children, the willingness to take booster shots, and trust in the efficiency of all vaccines. Resolving the aforementioned issues could be employed to improve the poor rate of vaccine administration in the area. For a more detailed insight, 70% of participants required further evidence to open their minds to booster shots; such hesitancy could be overcome by employing additional efforts in clarifying the misconceptions concerning booster shots as they are significantly affecting people’s willingness to take them.

One of the most prominent strengths that characterized our study is the large size of our sample. It was taken from a large university with students of different backgrounds from a broad spectrum of perspectives and beliefs. All of which are direct reasons to believe it to be representative of the attitudes of Jordan university students. Our interest in university students stems from the fact that Jordan’s population is mainly a young one, with the dominant age group being youth and adolescents. Additionally, our study manifested comprehensiveness in terms of the different topics it explored; all of which were recent issues directly related to vaccination in Jordan.

There are three potential limitations concerning the results of this study. Firstly, we used an online survey to collect responses from participants, which has always introduced potential biases that cannot be controlled for. Secondly, a more sophisticated model could be used to get a deeper understanding of the influential factors on vaccine choice. Thirdly and lastly, the ages of our study participants fell in a narrow range. Accordingly, we believe that recruiting individuals both younger and older than the ones who participated in the current study will increase inclusiveness and result in more representative findings.

If, as the present study suggests, the willingness to take the vaccine among young middle-aged university students is impressively high; then there is a need for research that explores other age groups’ willingness in order to specify which of them contributes the most to the unpleasant low rate of vaccination. Also, we believe that a comprehensive assessment of the current public health guidelines pointing out the fundamental defects in the system should be conducted for alterations and enhancements to follow. Finally, we think that it would be of considerable significance if future studies took the unexpected attitudes we captured in the current study and further explored them.

## 8. Conclusions

Vaccine uncertainty is one of the top global threats that hinders the fight to overcome any pandemic. Considering the drastically low rates of COVID-19 vaccination in Jordan, it was essential to investigate the perceptions and vaccine preferences to get a grasp of the possible contributing factors. Based on the conducted analysis, those studying medicine or health related majors, as well as those trusting in WHO guidelines, showed far more willingness to accept any type of vaccine available. Most participants believed the choice of vaccine type should be up to them. The majority of our sample showed uncertainty and required more evidence before considering booster shots. The inclusion of children in vaccination campaigns was a controversial topic, as only half of the participants indicated agreement. All in all, the dominant perception among students favored vaccine administration as a crucial step to evade a new COVID wave. With that being said, spreading knowledge using certified platforms, enhancing trust in WHO recommendations, updating individuals with the most recent studies and encouraging students to educate their families could provide a way out of the challenging anti-vaccination phenomenon.

## Figures and Tables

**Table 1:** Demographic characteristics (*n* = 417).

Characteristics	Frequency	Percentage (%)
Gender		
Female	289	69.3
Male	128	30.7
School Category		
Medical School	147	35.3
Non-Medical Health School	140	33.6
Non-Health School	130	31.2
Year of Study		
First Year	99	23.7
Second Year	192	46.0
Third Year	82	19.7
Fourth Year	28	6.7
Fifth Year	8	1.9
Sixth Year	8	1.9
**Age (Years)**	M = 20.14	SD = 1.683

**Table 2:** Vaccine Preferences.

Vaccine Choice	Frequency	Percent (%)
Pfizer	228	54.7
I don’t mind any vaccine	81	19.4
Sinopharm	59	14.1
I wouldn’t take any vaccine	26	6.2
Sputnik V	8	1.9
AstraZeneca	6	1.4
Any vaccine other than Sinopharm	5	1.2
Any vaccine other than mRNA vaccines	2	0.5
Johnson and Johnson’s	2	0.5

**Table 3:** Factors affecting vaccine preferences.

Vaccine	Associated Factors	*p* Value *	Effect Size (Interpretation) **
Pfizer	I prefer Pfizer because it is the most effective against new strains	<0.0001	0.567 (large effect)
	I prefer to avoid the Sinopharm vaccine as I believe it is ineffective	<0.0001	0.278 (small effect)
	I prefer the Pfizer vaccine as it allows me to travel to multiple countries	<0.0001	0.484 (medium effect)
Sinopharm	I prefer to avoid the AstraZeneca vaccine as I heard it causes strokes and heart attacks	<0.0001	0.257 (small effect)
	I prefer to avoid mRNA vaccines (such as pfizer) as I believe they have a lot of side effects	<0.0001	0.357 (medium effect)
Any vaccine	I trust the WHO (world health organization) standards, so I believe any approved vaccine is safe	<0.0001	0.423 (medium effect)
No vaccine	I believe COVID vaccines are unsafe and shouldn’t be taken	<0.0001	0.439 (medium effect)

Chi-square tests were used to determine the associations *, and the Cramer’s V effect size is shown for each association **.

**Table 4:** Attitudes towards recent issues in vaccination.

Question	Strongly Disagree	Disagree	Neutral	Agree	Strongly Agree
I believe vaccination is crucial to avoid a new wave from the new strains	5.3%	6.7%	17.0%	38.4%	32.6%
Hearing about the Indian (delta) strain encouraged me to take the vaccine	14.4%	21.3%	29.3%	22.8%	12.2%
I think children should be more involved in vaccination campaigns as the new strains affect them more commonly	7.7%	14.9%	32.9%	30.2%	14.4%
I believe vaccination is crucial to avoid a new wave from the new COVID-19 strains	5.3	6.7	24.9	42.9	15.3

## Data Availability

Data is available upon reasonable requests by emailing the communicating author.
